# Renal Vein Thrombosis Secondary to Pyelonephritis: Targeting a Thrombo-Inflammatory Entity

**DOI:** 10.3390/clinpract14030088

**Published:** 2024-06-09

**Authors:** Dimitris Kounatidis, Vasileios Papadimitropoulos, Natalia Vallianou, Aikaterini Poulaki, Krystalia Dimitriou, Ioanna Tsiara, Konstantinos Avramidis, Alexandra Alexopoulou, Dimitrios Vassilopoulos

**Affiliations:** 2nd Department of Internal Medicine, Hippokration University Hospital, National and Kapodistrian University of Athens, 11527 Athens, Greece; dimitriskounatidis82@outlook.com (D.K.); bpapad@hotmail.com (V.P.); krystalia_dim@hotmail.com (K.D.); avramidiskonst@gmail.com (K.A.); dvassilops@med.uoa.gr (D.V.)

**Keywords:** pyelonephritis, renal vein thrombosis, *Klebsiella pneumoniae*, thrombo-inflammation, percutaneous mechanical thrombectomy, xantho-granulomatous pyelonephritis

## Abstract

Renal vein thrombosis (RVT) is a relatively uncommon condition that is most frequently observed in individuals with nephrotic syndrome. While rare, pyelonephritis (PN) may serve as a predisposing factor for secondary RVT. In such cases, one should consider the possibility of RVT when patients fail to respond to appropriate antibiotic treatment. Typically, these patients require additional anticoagulation therapy for a duration of 3 to 6 months, with a generally favorable prognosis. In this report, we present the case of a 74-year-old female who developed RVT due to *Klebsiella pneumoniae* PN. Additionally, we reviewed 11 cases of PN complicated by RVT, which were documented in the PubMed database over a span of 40 years, emphasizing key elements in diagnostic and therapeutic approaches. Lastly, we elaborated upon the role of thrombo-inflammation, especially in the context of sepsis.

## 1. Introduction

Renal vein thrombosis (RVT) is a rare and potentially life-threatening condition, primarily observed in patients with nephrotic syndrome, owing to their underlying hypercoagulable state [1]. In neonates, RVT is one of the leading causes of venous thrombosis, often associated with severe dehydration or prolonged hypotension. If left untreated, RVT can lead to severe consequences, including extension into the inferior vena cava (IVC) and septic pulmonary embolism. Renal atrophy and papillary necrosis may also be observed [2].

RVT due to pyelonephritis (PN) has rarely been documented. To address this scarcity of information, we conducted a search on the PubMed database using the terms “Renal vein thrombosis and Pyelonephritis” in November 2023, identifying 15 related case reports. Four of these were excluded, with one involving a pediatric patient, one lacking an abstract, and two being written in French. Consequently, 11 case reports were included. Here, we present the case of a 74-year-old patient with poorly controlled diabetes mellitus, who developed RVT attributed to acute PN caused by *Klebsiella pneumoniae*. Furthermore, we will elaborate upon RVT as a consequence of PN in the context of the so-called “thrombo-inflammation” phenomenon.

## 2. Case Presentation

A 74-year-old female patient presented to the emergency department with a 3-day history of fever and chills. She also complained of dysuria, increased urinary frequency, and suprapubic pain over the past 3 weeks. Her medical history was notable for diabetes mellitus, hypertension, and hyperlipidemia, which were managed with metformin, vildagliptin, gliclazide, olmesartan, and simvastatin, respectively. Upon admission, the patient had a fever (39 °C), a pulse rate of 95/min, and a blood pressure of 105/60 mmHg. A clinical examination revealed tenderness in the right flank and renal angle (Giordano’s sign). Laboratory tests indicated a white blood count (WBC) of 10,480/mL, hyperglycemia (serum glucose 350 mg/dL), impaired renal function (urea 63 mg/dL and creatinine 1.6 mg/dL, GFR 34 mL/min/1.73 m^2^), and elevated C-reactive protein (CRP) at 116 mg/L (normal range < 5 mg/L). An arterial blood gas analysis ruled out diabetic ketoacidosis, while her HbA1c was 13.1%. The D-dimer levels were very increased at 4254 mg/L. A urinalysis showed a WBC of >100/hpf in urine (per high power field), a red blood cell (RBC) count of 5–6/hpf in urine, a pH of 7, and Specific Gravity (SG) of 1010, while the chest X-ray and abdominal ultrasound were normal. Blood and urine cultures were obtained, with the latter yielding a *Klebsiella pneumoniae* concentration of > 10⁵ cfu/mL (colony forming units/mL). The patient’s laboratory findings are listed in Table 1.

**Figure 1:**
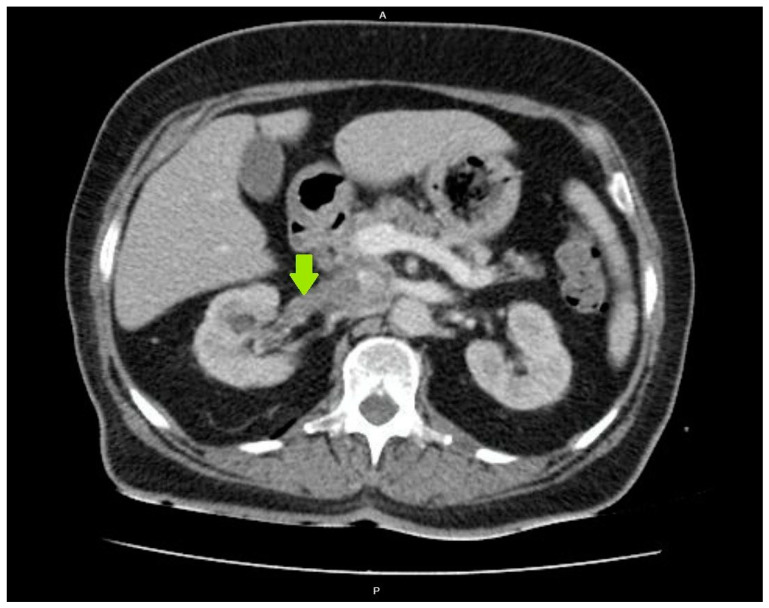
Renal vein thrombosis on a computed tomography (CT) scan of the abdomen.

The diagnosis of acute PN was clinically confirmed due to the presence of fever (39 °C) with chills and accompanying dysuria, increased urinary frequency, lower abdominal pain, and right flank pain with a positive Giordano sign, while the laboratory findings indicated increased CRP levels of 116 mg/L (upper normal limit 5 mg/L) and a WBC in urine sediment of >100/hpf (per high power field), and the urine culture yielded a *Klebsiella pneumoniae* concentration of >10⁵ cfu/mL. As the diagnosis of acute PN was established, the patient received an empirical antimicrobial treatment with piperacillin–tazobactam, and intravenous fluids and insulin were administered as well. Despite this treatment, the patient’s condition did not improve, showing a persistent fever and high inflammatory markers. Therefore, on the fourth day, an abdominal CT scan was performed to rule out the formation of a renal abscess, which revealed right renal pelvis and calyceal dilatation, as well as RVT extending into the inferior vena cava (Figure 1). A chest CT scan and echocardiography did not show any signs of pulmonary embolism, and both CT and magnetic resonance imaging (MRI) of the abdomen excluded the existence of concomitant renal cell carcinoma.

Thrombophilia screening, which included tests for proteins C and S, antithrombin deficiency, factor V Leiden, prothrombin 20210A, as well as antiphospholipid antibodies, yielded normal results. The patient was homozygous for the MTHFR (C677T) mutation, but the serum homocysteine levels were within the normal range. A 24 h urine albumin measurement did not align with nephrotic syndrome. Antibiotic treatment was continued intravenously for 14 days, followed by 14 more days of oral ciprofloxacin. Piperacillin–tazobactam was chosen initially, as the patient had diabetes mellitus and we initially intended to have coverage against *Pseudomonas aeruginosa* as well. We administered piperacillin–tazobactam iv for 14 days, and after discharge from the hospital, we administered ciprofloxacin to the patient for another 14 days. Ciprofloxacin was administered according to the results of susceptibility to various antibiotics. As this was a case of complicated PN in the context of thrombo-inflammation, and as such cases with RVT had been treated with antibiotics for 4–6 weeks in the medical literature, we also decided to administer antibiotics for such a long period of time, i.e., 4 weeks, totally.

Additionally, anticoagulant therapy with low-molecular-weight heparin was initiated, as the patient’s expected poor compliance with oral treatment precluded the use of acenocoumarol. One month later, the patient was afebrile, in good clinical condition, and scheduled for a reassessment in approximately 2 months.

## 3. Discussion

### 3.1. Clinical Presentation and Treatment of PN

PN represents a complicated urinary tract infection that develops as uropathogens ascend to the kidneys, or in cases of renal seeding, in the setting of bacteremia. The most common causative bacteria are *Enterobacterales*, with *Escherichia coli*, *Klebsiella* spp., and *Proteus* spp. being the primary offenders. Other pathogens, such as *Pseudomonas aeruginosa*, *Staphylococcus aureus*, and *Enterococci*, may also be responsible [3]. Clinically, PN manifests with fever, chills, flank pain, and costovertebral angle tenderness. In some instances, patients might experience gastrointestinal symptoms like nausea, vomiting, epigastric or lower abdominal pain, and diarrhea. Renal or perinephric abscesses are the most common complications, while patients with diabetes may potentially develop emphysematous PN and papillary necrosis. Treatment typically involves antibiotics, with beta-lactams, fluoroquinolones, and carbapenems as the usual therapeutic options. As there is a substantial increase in the prevalence of extended-spectrum beta lactamase (ESBL)-producing bacteria worldwide, urinary and blood cultures with susceptibility testing are needed to guide antimicrobial chemotherapy. Cases such as hospitalization or if a patient has attended a health care facility in the past three months as well as had a prior administration of fluoroquinolones, trimethoprim-sulfamethoxazole, or beta lactam antibiotics during the past three months should raise suspicion of the existence of a resistant urinary microorganism. In addition, recent travel in places with a high prevalence of resistant bacteria, such as India, or a personal history of a resistant bacterium should be considered when antibiotic coverage is administered. Moreover, if a patient is severely ill, the administration of broad-spectrum antibiotics with a plausible step-down decision according to susceptibility testing, when this is available, is advisable [4,5,6].

### 3.2. Clinical Presentation of RVT

RVT is a potentially fatal vascular complication characterized by the formation of thrombi in the renal vein(s) or their branches. The exact prevalence of RVT in adults remains unclear, as a significant number of cases are only discovered incidentally through imaging studies or when they lead to severe adverse effects, such as pulmonary embolism [7]. Symptomatic RVT cases often present with symptoms related to renal infarction, including flank pain, hematuria, and sometimes fever, making it challenging to differentiate from acute PN. A marked elevation in serum lactate dehydrogenase is a typical laboratory finding, but not pathognomonic. Bilateral RVT may lead to acute kidney injury, gross hematuria, and heavy proteinuria due to renal vein obstruction and the absence of collateral venous return [8].

RVT predominantly occurs in patients with nephrotic syndrome, particularly in cases of membranous nephropathy, likely because of the loss of anticoagulant proteins (antithrombin and plasminogen) in the urine. Other risk factors include renal cell carcinoma, inherited procoagulant defects, trauma, renal transplantation, oral contraceptives, extrarenal compression of the renal vein, and paroxysmal nocturnal hemoglobinuria [9]. In extremely rare cases, PN serves as the underlying cause of RVT. In the case we present, the patient suffered from PN due to *K. pneumonia*, leading to right RVT that extended into the inferior vena cava. To date, only a small number of similar cases have been documented in the literature. The clinical characteristics of these patients are described in Table 2 [10,11,12,13,14,15,16,17,18,19,20].

Most cases involved females (63.6%), with a median patient age of 49.7 years, while diabetes mellitus was present in four patients (Table 2). Thrombophilia screening revealed hyperhomocysteinemia in just one case (patient 8). *Enterobacterales* were the infectious agents in nine cases, with *K. pneumoniae* and *E. coli* being implicated in five and four cases, respectively. In two patients, *S. aureus* was isolated. Most cases affected the right kidney (63.6%), with one patient experiencing bilateral RVT. This contradicts the prevailing knowledge that RVT is more common on the left kidney, likely due to the longer left renal vein compared to the right. In 9 out of 11 cases, thrombosis extended into the inferior vena cava, and two patients developed septic pulmonary embolism. Most patients responded successfully to antibiotic and anticoagulant treatments, including low-molecular-weight heparin (LMWH) or warfarin. Three patients underwent nephrectomy, and in one case, percutaneous embolectomy was performed. Follow-up assessments using a CT scan, conducted after 3 to 6 months, revealed RVT recanalization in all cases. Notably, patient 3 (Table 2) suffered from emphysematous PN, a condition traditionally seen among patients with diabetes.

In addition to the documented cases, three case reports in the literature describe patients with RVT resulting from xanthogranulomatous pyelonephritis (XGP). XGP is a rare form of chronic PN characterized by the infiltration of the renal parenchyma by lipid-laden macrophages or xanthoma cells. Most cases are linked to chronic urinary tract obstruction and infection, typically caused by *Enterobacterales*. The “bear’s paw” sign on CT imaging is a distinctive and pathognomonic radiographic finding. However, XGP may often be mistaken for renal cell carcinoma, and nephrectomy is sometimes needed to establish the diagnosis of XGP or renal cell carcinoma, as the differential diagnosis between the two clinical entities may be difficult without histopathological findings. In addition, nephrectomy is usually the preferred treatment modality in patients with XGP [21,22,23].

### 3.3. The Role of Infection in Thrombosis: Thrombo-Inflammation

**Figure 2:**
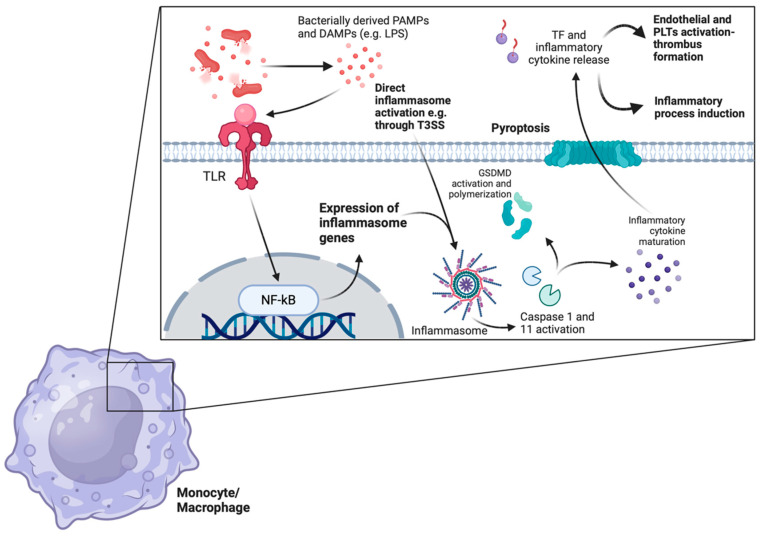
TF is released during sepsis in an inflammasome-dependent way. Bacterially derived DAMPs and PAMPs are recognized by several monocytic or macrophage TLRs. Recognition leads to the downstream expression of several inflammasome-related genes through the activation of the NF-kB pathway. Inflammasome formation cleaves the procaspase-1 peptide to the active caspase-1 protein in the classical inflammasome pathway, which eventually leads to the maturation of several proinflammatory cytokines and GSDMD activation, polymerization, and pyroptosis through membrane pore formation. Of note, LPS directly activates inflammasomes and leads to non-classical caspase-11-induced pyroptosis. Additionally, both LPS itself as well as TLR-mediated monocyte/macrophage priming leads to increased TF expression on plasma membranes. The subsequent release of TF-rich microvesicles into the circulation boosts endothelial and platelet activation, potentiating inflammatory thrombus formation. (TF: tissue factor, DAMPs: danger-associated molecular patterns, PAMPs: pathogen-associated molecular patterns, TLRs: Toll-like receptors, GSDMD: gasdesmin-D, LPS: lipopolysaccharide, T3SS: type III secretion system, PLTs: platelets.) The image was created on biorender.com.

**Figure 3:**
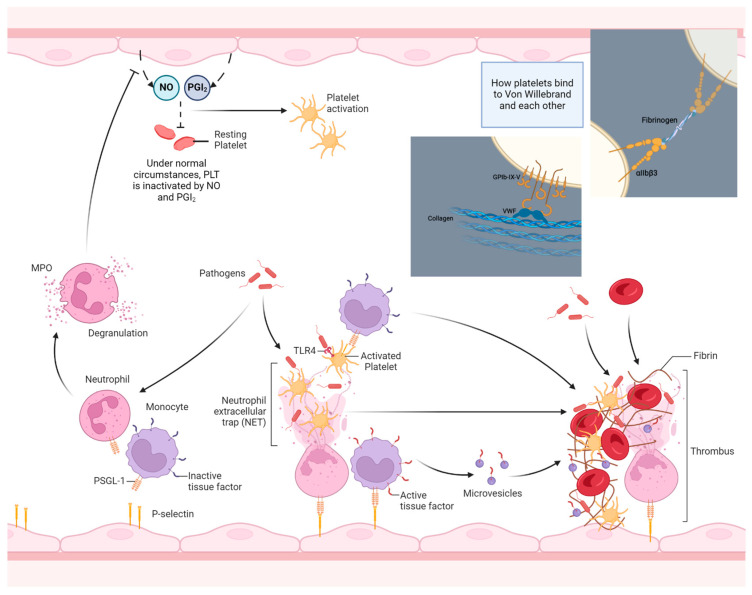
An overview of the thrombo-inflammation process. During inflammation, bacterial products per se as well as pro-inflammatory cytokines, such as TNF-a, activate endothelial cells to express P-selectins. Meanwhile, pathogens in the bloodstream and in tissues induce WBC activation, leading to the increased surface expression of PSGL-1, among others. After binding to endothelial selectins and after contact with pathogens (or pathogen-derived PAPMs/DAMPs), neutrophils experience cellular death through netosis, and NETs bind and activate bystander PLTs acting as scaffolds for thrombus formation. Meanwhile, activated monocytes express increased amounts of membrane-bound TF, which is subsequently released in the form of active TF microvesicles in the circulation after their activation or death (see also Figure 3). Additionally, the degranulation of neutrophils upon activation, especially MPO, degrades the NO excreted from resting endothelium and potentiates PLT activation and thrombus formation. (PSGL-1: P-selectin glycoprotein ligand, DAMPs: danger-associated molecular patterns, PAMPs: pathogen-associated molecular patterns, TLRs: Toll-like receptors, NO: nitric oxide, PGI2: prostaglandin I2, vWF: von Willebrand factor.) The image was created on biorender.com.

Despite the fact that thrombosis and inflammation are two distinct entities, they are also interconnected. Notably, there is an intricate interplay between thrombosis and inflammation, which leads to the so-called phenomenon of “thrombo-inflammation” [24,25,26,27]. Thrombo-inflammation may result from severe infection, especially in the course of sepsis, but it may be the consequence of a non-infectious inflammatory process as well [24,25]. In sepsis, immune thrombotic dysregulation may occur, which may lead to disseminated intravascular coagulation (DIC), i.e., thrombotic complications in the microcirculation throughout the human body. Apart from platelets, other cells, such as neutrophils, monocytes, and macrophages, are deeply implicated in this process [24,25,26]. More specifically, platelets and neutrophils recognize bacteria via their pattern recognition receptors (PRRs), mainly Toll-like receptors (TLRs). Platelets have the capacity to migrate inside vessels. This migratory ability is mediated by the cytoskeleton protein actomyosin as well as by the interaction of aIIbβ3 integrin with fibrinogen [23,24,25,26,27,28,29]. Integrins are a family of at least 24 members, which promote cell adhesion, migration, and proliferation [28,29]. These molecules, which contain different combinations of alpha and beta chains, consist of extracellular domains, a membrane-spanning domain, and an intracellular domain or “tail” of the integrins. Integrins have the potential to serve as bidirectional signaling pathways. Their extracellular domains bind to fibronectin, fibrinogen, intercellular adhesion molecules (ICAMs), and complement [27,28,29]. The most abundant integrin in platelets is the aIIbβ3 integrin, which was previously known as the GPIIb/IIIa complex [27,28,29]. This integrin, when activated, undergoes a conformational change and binds to fibrinogen, fibrin, and von Willebrand factor (vWF), thus resulting in platelet aggregation [24,25,26,27,28,29]. Apart from their accumulation, platelets, mainly via TLRs, are suggested to bind to bacteria and present them to neutrophils, which are then activated to form neutrophil extracellular traps (NETs) [26,30,31]. NETs are composed of neutrophils as well as nuclear DNA, histones, and neutrophil-derived granules, which release proteins, such as neutrophil elastase, cathepsin G, and myeloperoxidase. Neutrophil elastase and cathepsin G are serine proteases released by the azurophil granules of neutrophils, which exhibit antibacterial properties. In addition, they are both involved in platelet function and thrombus formation [31,32]. They are also considered to be danger-associated molecular patterns (DAMPs), which participate in the activation of endothelial cells and the release of vWF [31,32]. On the other hand, myeloperoxidase, which is secreted by azurophil granules as well as a DAMP, has been suggested to be implicated in endothelial dysfunction by decreasing the availability of nitric oxide in endothelial cells. Additionally, myeloperoxidase binds directly to platelets, leading to platelet aggregation [31,32]. Regarding NETs, it has been demonstrated that not every neutrophil may be involved in NET formation. It is noteworthy that older neutrophils, compared with neutrophils that are newly released from bone marrow, have the tendency to be implicated in NET formation [31,32]. In addition, S100A8/A9, which is an alarmin also known as calprotectin, is the main cytosolic protein bound to NETs. The family of alarmins includes peptides, such as interleukins, which are released in response to infection, and they are being implicated in various immune responses to infection. S100A8/A9, which is also considered a DAMP, exerts antibacterial and prothrombotic features as well. It is expressed in neutrophils, monocytes, and platelets. S100A8/A9, via the A9 subunit, exhibits high affinity to glycosaminoglycans of the glycocalyx of bacteria. Based on the aforementioned observation, an inhibitor of S100A9, named paquinimod, has lately been used in experimental models of sepsis as an agent with anti-inflammatory properties [32,33]. As already mentioned above, only activated platelets have the propensity to be involved in the formation of NETs. The pathogenesis of RVT related to PN is thought to be linked to the ability of Gram-negative bacteria to provoke thrombosis through endotoxin production. Endotoxins are lipopolysaccharides (LPSs) exclusively found in the outer membrane of Gram-negative bacteria, in sharp contrast to Gram-positive bacteria, which lack LPSs [32,33]. Notably, LPS has the potential to provoke the formation of NETs [32,33]. The upregulation of the coagulation cascade is triggered by LPS binding to Toll-like receptors (TLRs), leading to endothelial cell and platelet activation. Histones, which represent 70% of the proteins’ NETs composition, bind to TLRs, especially TLR2 and TLR4, leading to an enhancement in thrombotic stages [32,33,34]. Histones bind to platelets, resulting in platelet activation and accumulation, as well as thrombin formation. In this thrombo-inflammatory milieu, the increased expressions of procoagulant molecules, such as tissue factor (TF) and ICAM, together with a decreased expression of thrombomodulin, all contribute to thrombogenesis, partly by a TLR2 and TLR4 pathway [32,33,34]. Regarding TF, it has been suggested that it is highly expressed by monocytes in the context of sepsis [32,33,34,35,36,37,38]. Notably, LPS via the non-canonical inflammasome activation pathway, i.e., mediated by the activation of caspase-11, may lead to the gasdermin-D-dependent formation of membrane cell pores. This process results in a form of regulated cell death called pyroptosis. It has been proposed that pyroptosis is responsible for the release of TF by monocytes as well as by macrophages. While LPS provokes non-canonical inflammasome activation and the subsequent release of TF by monocytes, the type III secretion system (T3SS) of bacteria results in a caspase-1 activation of the inflammasome, i.e., the canonical activation of the inflammasome and increased TF expression [32,33,34,35,36,37,38]. Therefore, the inflammasome seems to play a crucial role in the increased expression of TF by monocytes and macrophages. Figure 2 and Figure 3 depict the involvement of the inflammasome in the thrombo-inflammation process.

Additionally, hyperglycemia might be implicated in thrombus formation, particularly in patients with poorly controlled diabetes mellitus. Elevated serum glucose levels increase platelet activation while enhancing the effects of procoagulants and antifibrinolytic proteins. Furthermore, reduced levels of anticoagulants, such as protein C and thrombomodulin, are observed [24,25].

### 3.4. Diagnosis and Management of RVT

Diagnosing RVT can be challenging since a significant portion of cases are asymptomatic. Patients with nephrotic syndrome should be evaluated for RVT if they experience new-onset flank or generalized abdominal pain, gross hematuria, or an unexplained deterioration of renal function. RVT should also be considered in cases of PN that do not respond to appropriate therapy, as a delayed diagnosis can have devastating consequences. In the clinical setting, if a patient does not improve during the first few days of administration of the correctly selected antibiotic (i.e., according to susceptibility testing or/and the regional resistance rates taken into consideration), a CT scan should be performed with intravenous contrast material in order to rule out adverse effects of PN, such as the formation of an abscess or the presence of RVT. Inferior vena cava venography and subsequent renal vein venography is the gold standard method for diagnosing RVT. However, as renal vein venography is an invasive procedure, which needs experienced personnel to be performed, in the clinical setting, CT angiography (CTA) is the preferred method for initial diagnosis. CTA exhibits sensitivity and specificity rates of approximately >95%. When CTA is unavailable, gadolinium-enhanced MR venography (MRV) may serve as an alternative. MRV’s sensitivity and specificity is reported to be only slightly inferior to CTA [39]. It is noteworthy that Doppler ultrasonography is less sensitive than CTA or MRV and should only be used when CTA or MRV cannot be performed or as an adjunctive and useful method for diagnosis [40,41]. In particular, ultrasonographic signs are the enlargement of the affected kidney(s), while Doppler ultrasonography may, in addition, reveal disturbed flow in the renal vein(s). Doppler ultrasonography is being widely used for the detection of RVT among patients who have undergone kidney transplantation, as in this particular category of patients, it has been associated with high sensitivity rates [40,41]. With the advent of 18 FDG PET/CT scan (18 fluorodeoxyglucose positron emission tomography/computed tomography), which is now more widely used, more information about the benign or malignant nature of RVT may be obtained [42,43,44]. Due to the fact that RVT may develop in the context of renal cell carcinoma, the metabolic state of the thrombus in renal vein(s) in the PET/CT scan may shed light on whether the origin of the thrombus is benign or not [42,43,44].

In the past, nephrectomy was the primary treatment for RVT. However, contemporary practice leans towards conservative management consisting of anticoagulation therapy with a transition from LMWH to vitamin K antagonists [45]. Limited data are available on the use of direct thrombin inhibitors (e.g., dabigatran and argatroban) and factor Xa inhibitors (e.g., rivaroxaban, apixaban, and fondaparinux) in patients with RVT, so their administration remains controversial [46,47,48,49,50,51,52]. However, there has recently been an increasing number of reports advocating their use among patients with RVT [46,47,48,49,50,51,52]. In general, there are many reports supporting their use even in cases of deep venous thrombosis of atypical locations [50,51,52]. Furthermore, in circumstances unresponsive to heparin, thrombolysis using local infusion of recombinant tissue plasminogen activator (rTPA) may be a life-saving treatment modality, particularly in patients with a massive clot burden or bilateral RVT leading to acute renal failure. Surgical thrombectomy or percutaneous mechanical thrombectomy is a recommended approach when thrombolysis is contraindicated and may be life-saving as well [41,53].

A recent retrospective study by Zhang et al. suggests that combining anticoagulant treatment with an endovascular intervention may be more effective than using anticoagulants alone in the acute phase of isolated RVT. An endovascular intervention includes catheter-directed thrombolysis and mechanical thrombectomy, with beneficial outcomes related to improved renal function and the prevention of RVT progression [54].

In cases of RVT secondary to PN, patients should receive antibiotics for 4 to 6 weeks, while the duration of anticoagulant therapy typically spans 3 months [49]. Nonetheless, the use of vitamin K antagonists for 6 months is a reasonable choice, as VKAs are typically administered for a minimum of 6 to 12 months in RVT cases [55,56,57]. RVT is a potentially fatal clinical entity if left untreated. However, as shown in Table 2, among the 11 cases of PN and RVT, 10 cases had favorable outcomes, whereas there was no information regarding the last case. Therefore, we conclude that the timely diagnosis of RVT in the context of PN is of the utmost importance in the clinical setting.

## 4. Conclusions

RVT is an exceptionally rare complication of acute pyelonephritis. The so-called phenomenon of “thrombo-inflammation” is the result of an intricate interplay between pro-inflammatory factors and the immune system response, leading to thrombus formation. In this complex process, the roles of platelet activation, NET formation, and inflammasome involvement seem to be of the utmost importance. The early recognition of RVT in cases of PN is crucial, as untreated RVT carries a mortality rate exceeding 50%. Current treatment protocols support a conservative approach, including the use of antibiotics and anticoagulants. Apart from LMWH and the switch to vitamin K antagonists, newer anticoagulants, such as direct oral anticoagulants (DOACs), are being increasingly used. Some experts advocate adjunctive endovascular intervention, which may lead to more favorable outcomes. However, further large-scale studies are necessary to establish recommendations regarding the optimal treatment of these patients.

## Figures and Tables

**Table 1:** Main laboratory findings.

Laboratory Parameter	Normal Range	Upon Admission	Upon Discharge
WBC	4000–11,000/mL	10,480/mL	5000/mL
Hb	12–18 g/dL	10 g/dL	11.1 g/dL
PLTs	150,000-400,000/mm^3^	413,000/mm^3^	392,000/mm^3^
APTT	27–35 s	27 s	26.5 s
Fibrinogen	200–400 mg/dL	506 mg/dL	393 mg/dL
D-dimers	<500 μg/L	4254 μg/L	1654 μg/L
HbA1c	<6.5%	13.1%	-
Serum glucose	80–130 mg/dL	174 mg/dL	104 mg/dL
Serum urea	18–55 mg/dL	63 mg/dL	42 mg/dL
Serum creatinine	0.72–1.25 mg/dL	1.5 mg/dL	1.0 mg/dL
Serum sodium	136–145 mmol/L	136–145 mmol/L	143 mmol/L
Serum potassium	3.5–5.1 mmol/L	5.5 mmol/L	4.5 mmol/L
SGOT	5–34 U/L	17 U/L	27 U/L
SGPT	0–55 U/L	23 U/L	24 U/L
ALP	40–150 U/L	33 U/L	32 U/L
g-GT	12–64 U/L	28 U/L	34 U/L
Total bilirubin	0.20–1.20 mg/dL	0.60 mg/dL	0.30 mg/dL
Serum albumin	3.5–5 g/dL	3.5 g/dL	3.5 g/dL
CRP	<5 mg/L	116 mg/L	19 mg/L

**Table 2:** Clinical characteristics of 11 published case reports.

Case	Author/ Year	Gender/ Age (Years Old)	DM/ Thrombophilia	Culture	Site of Thrombosis	IVC/ PE	Treatment	Follow-Up/ Resolution of Thrombosis
1	Ben Hassine/ 2023	Female/ 29	Yes/ No	*Klebsiella pneumoniae*	Left	No/ No	Antibiotics, LMWH, warfarin	6 months/ Yes
2	Cedeira/ 2020	Female/ 38	No/ No	*MRSA*	Left	Yes/ Yes (Septic Emboli)	Antibiotics, LMWH, warfarin, bilateral nephrectomy	NS/ yes
3	Jain/ 2019	Female/ 32	No/ No	*Escherichia coli*	Right	Yes/ NS	Antibiotics, heparin, nephrectomy	3 months/ yes
4	Assimakopoulos/ 2018	Male/ 71	Yes/ No	*Escherichia coli*	Right	Yes/ NS	Antibiotics, LMWH, acenocoumarol	3 months/ yes
5	Lurz/ 2018	Female/ 35	No/ No	*Klebsiella pneumoniae*	Right	Yes/ NS	Antibiotics, LMWH, warfarin	6 months/ yes
6	Yildiz/ 2016	Female/ 68	No/ No	*Escherichia coli*	Right	No/ NS	Antibiotics, LMWH, warfarin	6 weeks/ yes
7	Harris/ 2012	Female/ 62	Yes/ No	*Klebsiella pneumoniae*	Bilateral	No/ Yes (Septic Emboli)	Antibiotics, LMWH, warfarin	4 weeks/ resolution of the right thrombosis
8	Kumar/ 2009	Male/ 59	No/ Yes	*Escherichia coli*	Left	Yes/ No	Antibiotics, LMWH, warfarin	3 months/ yes
9	Novelli/ 2007	Male/ 50	NS/ NS	*MSSA*	Right	Yes/	Antibiotics, percutaneous embolectomy	6 months/ yes
10	Bassilios/ 2004	Female/ 45	Yes/ No	*Klebsiella pneumoniae*	Right	Yes/ NS	Antibiotics, LMWH, warfarin	6 months/ yes
11	Eijsten/ 1986	Male/ 58	NS/ NS	*Klebsiella pneumoniae*	Right	Yes/ NS	Antibiotics, nephrectomy	NS/ NS

Abbreviations: DM: Diabetes Mellitus, MRSA: *Methicillin-Resistant Staphylococcus aureus,* MSSA: *Methicillin-Sensitive Staphylococcus aureus*, IVC: Inferior Vena Cava, PE: Pulmonary Embolism, LMWH: Low-Molecular-Weight Heparin, NS: Not Specified.

## Data Availability

Data are available from the corresponding author upon reasonable request.

## List of Abbreviations

CTA: Computed Tomography Angiography; DAMPs: Damage-Associated Molecular Patterns; DIC: Disseminated Intravascular Coagulation; DOACs: Direct Oral Anticoagulants; ESBL: Extended-Spectrum Beta Lactamases; IVC: Inferior Vena Cava; LMWH: Low-Molecular-Weight Heparin; MRV: Magnetic Resonance Venography; NETs: Neutrophil Extracellular Traps; PET/CT: Positron Emission Tomography/Computed Tomography; PN: Pyelonephritis; PRR: Pattern Recognition Receptors; RVT: Renal Vein Thrombosis; TF: Tissue Factor; T3SS: Type III Secretion System; TLRs: Toll-Like Receptors; rTPA: Recombinant Tissue Plasminogen Activator; vWF: von Willebrand Factor; WBC: White Blood Count; XPG: Xanthogranulomatous Pyelonephritis.
